# Epidemiology and Predictors of 90-Day Mortality in Patients with *Clostridioides difficile* Infection: A Retrospective Single-Center Study from Galati, Romania

**DOI:** 10.3390/microorganisms14102261

**Published:** 2026-10-05

**Authors:** Pantelie Nicolcescu, Gabriel-Valeriu Popa, Mădălina-Nicoleta Matei, Alina Plesea-Condratovici, Alina-Viorica Iancu, Manuela Arbune

**Affiliations:** Medicine and Pharmacy Faculty, Research Centre in the Medical-Pharmaceutical Field, “Dunărea de Jos” University of Galați, 800010 Galați, Romania; pantelie.nicolcescu@ugal.ro (P.N.); madalina.matei@ugal.ro (M.-N.M.); manuela.arbune@ugal.ro (M.A.)

**Keywords:** *Clostridioides difficile* infection, healthcare-associated infection, epidemiological surveillance, 90-day mortality, Romania

## Abstract

*Clostridioides difficile* infection (CDI) is associated with substantial morbidity and mortality, but data on 90-day mortality after hospitalization from Romania remain limited. This study characterized hospitalized patients with CDI at a regional infectious diseases hospital in south-eastern Romania and evaluated factors associated with 90-day all-cause mortality. We conducted a retrospective observational study of adults with laboratory-confirmed CDI admitted to Infectious Diseases Clinical Department I between 1 January and 31 December 2024. The date of hospital admission for the index CDI episode was defined as time zero for the 90-day follow-up. Clinical, epidemiological, laboratory, and treatment variables were analyzed; ATLAS components were assessed at CDI diagnosis, while urinary-catheter (UC) exposure was analyzed separately because the source records did not reliably establish whether catheter placement preceded or followed CDI diagnosis in every patient. Associations with 90-day all-cause mortality were assessed using multivariable logistic regression. Among 170 patients (median age, 70 years), 55 died within 90 days (32.4%; 95% CI, 25.4–39.9%). Overall, 99/170 patients (58.2%; 95% CI, 50.4–65.7%) had an age-adjusted Charlson Comorbidity Index >5. In the final multivariable model, ATLAS score >4 (adjusted OR [aOR], 3.52; 95% CI, 1.56–7.95; *p* = 0.002) and indwelling UC exposure (aOR, 2.88; 95% CI, 1.12–7.37; *p* = 0.028) were associated with 90-day all-cause mortality. The model showed good discrimination (AUC, 0.746; 95% CI, 0.664–0.828). Ninety-day all-cause mortality was substantial in this single-center Romanian cohort. Higher ATLAS score and UC exposure were associated with mortality, but the UC association is exploratory because catheter timing relative to CDI diagnosis was not reliably available. Prospective multicenter external validation is required before these findings are used for clinical risk stratification.

## 1. Introduction

Healthcare-associated infections (HAIs) remain a major challenge for healthcare systems worldwide, contributing substantially to patient morbidity, mortality, and healthcare utilization. Despite considerable advances in infection prevention and control, the increasing complexity of medical care, population aging, the widespread use of antibiotics, and the growing burden of antimicrobial resistance have sustained HAIs as a major global public health concern. The World Health Organization (WHO) recognizes the prevention of healthcare-associated infections as a strategic priority for improving patient safety and reducing the global burden of nosocomial infections [1].

Within this context, *Clostridioides difficile* infection (CDI) is an important healthcare-associated infection associated with substantial morbidity, prolonged hospitalization, recurrence, and mortality. Although CDI has traditionally been considered predominantly hospital- and antibiotic-associated, epidemiological changes over recent decades have highlighted the contribution of community-associated infections and the need for continuous epidemiological surveillance [2,3,4]. Previous antibiotic exposure remains one of the major recognized risk factors for CDI, while advanced age and a high burden of comorbidities contribute to severe disease and adverse outcomes [3,4,5,6].

Recent advances have been accompanied by important conceptual changes in the classification and surveillance of CDI. The taxonomic reclassification of *Clostridium difficile* as *Clostridioides difficile*, the adoption of standardized epidemiological definitions for healthcare-associated and community-associated infection, and the refinement of the concept of recurrent CDI have contributed to a more consistent framework for epidemiological surveillance and clinical research [5,6,7,8]. Current expert recommendations further emphasize that, in the absence of molecular strain typing, clinically recurrent episodes should be classified as recurrent CDI (rCDI), whereas differentiation between relapse and reinfection should be reserved for molecular epidemiological investigations [7,8].

In Romania, CDI remains a major challenge for healthcare institutions, particularly in the context of high antibiotic consumption, an aging population, and the increasing prevalence of patients with multiple comorbidities. National surveillance data and recent Romanian studies indicate that CDI continues to be among the most frequently reported healthcare-associated infections, with an increasing incidence following the COVID-19 pandemic and a persistently high prevalence of prior antibiotic exposure among affected patients [9,10]. However, comparable Romanian data on 90-day post-discharge mortality and its predictors remain scarce, limiting direct national benchmarking of the present findings.

Although in-hospital mortality remains an important outcome in patients with CDI, it may underestimate the overall mortality burden, as it does not capture deaths occurring after discharge. Post-discharge mortality may be influenced by recurrent infection, persistent frailty, treatment-related complications, and decompensation of underlying comorbidities. Consequently, 90-day all-cause mortality provides a more comprehensive assessment of short-term prognosis and may better capture the clinical burden of CDI beyond the index hospitalization [6,11,12].

Accordingly, this study aimed to characterize the clinical and epidemiological profile of patients with CDI treated at a single regional infectious diseases hospital in Galați, south-eastern Romania, and to evaluate factors associated with 90-day all-cause mortality. The study was not designed to provide nationally representative estimates.

## 2. Materials and Methods

### 2.1. Study Design and Setting

We conducted a retrospective observational study at the “Sf. Cuv. Parascheva” Clinical Hospital of Infectious Diseases, Galați, Romania, a 160-bed specialized infectious diseases hospital without an intensive care unit. The hospital serves Galați County and receives referrals from adjacent areas in south-eastern Romania, representing a mixed urban–rural regional catchment population. Because this was a single-center study in a specialized referral hospital, the findings should not be interpreted as representative of Romania as a whole. The study included all eligible patients admitted to Infectious Diseases Clinical Department I between 1 January and 31 December 2024.

The study was approved by the hospital’s Ethics Committee (Approval No. 50/23.07.2026).

### 2.2. Study Population

During the study period, the hospital recorded 4373 admissions, of which 1652 were to Infectious Diseases Clinical Department I and 2721 to other hospital departments. Among patients admitted to Department I, 254 records carried a primary or secondary discharge diagnosis coded as ICD-10 A04.7 (Enterocolitis due to *Clostridioides difficile*). After application of the predefined clinical and microbiological eligibility criteria, 63 records were excluded because they did not fulfill the study case definition for CDI, leaving 191 patients eligible for the 90-day follow-up analysis. Reasons for not fulfilling the case definition included GDH-positive/toxin A/B-negative results without alternative confirmatory evidence of pseudomembranous colitis and documentation of *C. difficile* colonization without compatible clinical symptoms. Of the 191 eligible patients, 21 lacked documented written informed consent authorizing participation/data use and were excluded, leaving a final analytic cohort of 170 patients. Patients were included irrespective of sex, body weight, demographic characteristics, comorbidities, co-infections, concomitant medications, or previous vaccination status. These characteristics were recorded as clinical or epidemiological variables when available and were considered in the subsequent analyses.

**Figure 1:**
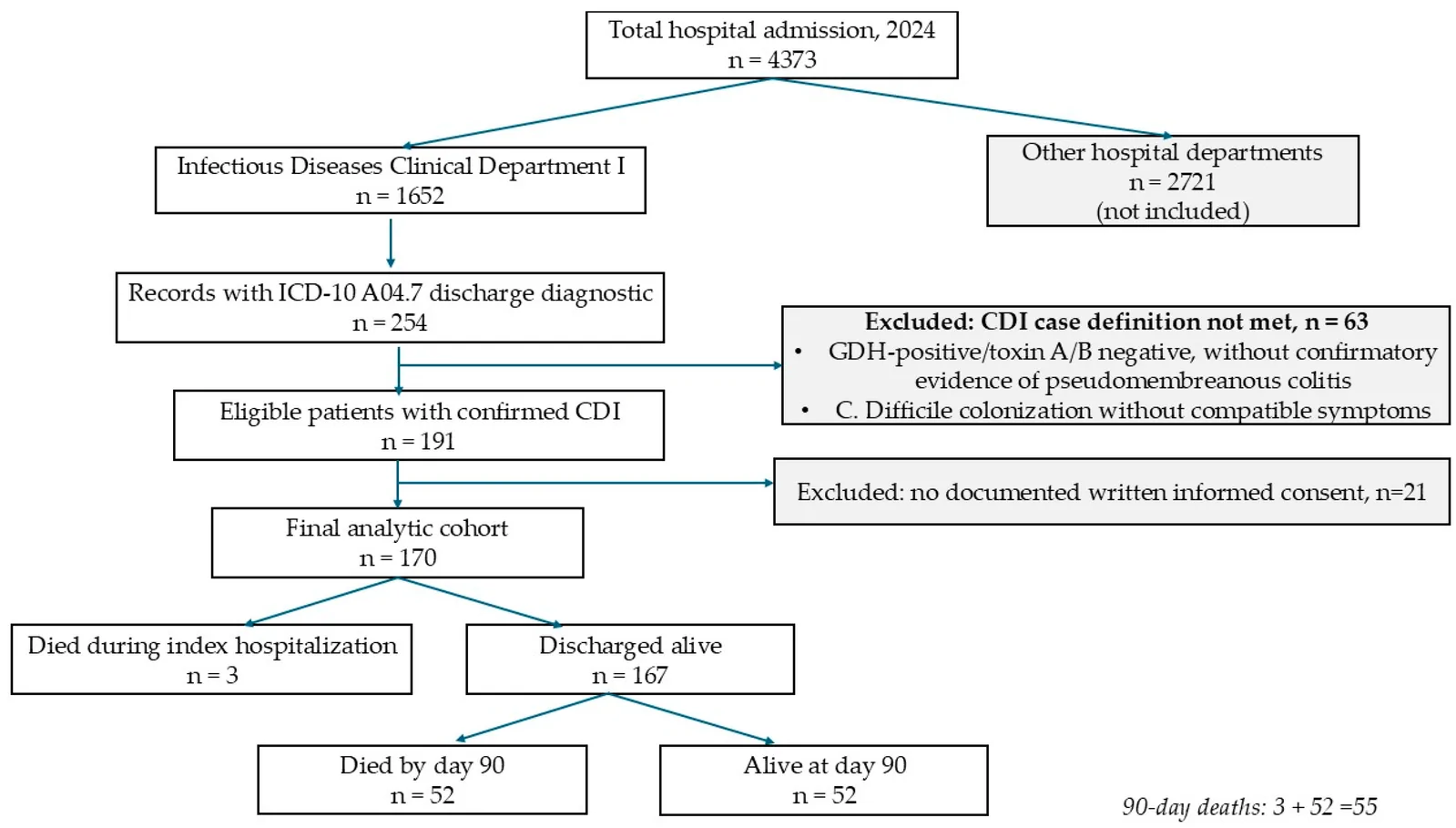
Study flow diagram. Of 4373 total hospital admissions during 2024, 1652 were to Infectious Diseases Clinical Department I and 2721 to other hospital departments. Among Department I admissions, 254 records carried an ICD-10 A04.7 discharge diagnosis; 191 fulfilled the predefined CDI case definition and were eligible for 90-day follow-up. Sixty-three records were excluded because they did not fulfill the case definition, including GDH-positive/toxin A/B-negative results without confirmatory evidence of pseudomembranous colitis and *C. difficile* colonization without compatible clinical symptoms. Of the 191 eligible patients, 21 lacked documented written informed consent and were excluded, leaving a final analytic cohort of 170 patients (3 in-hospital deaths and 167 discharged alive). All 167 patients discharged alive were followed to day 90; 52 died during follow-up and 115 were alive at day 90.

The predefined exclusion criteria were age <18 years, failure to fulfill the clinical and microbiological criteria for CDI, or absence of documented written informed consent authorizing the use of personal data for research and medical education purposes, in accordance with institutional policies and local procedures (Figure 1).

### 2.3. Definitions

In the present study, CDI was defined in accordance with ECDC/CDC-aligned criteria [5,6] as compatible clinical disease (at least three unformed stools within 24 h, ileus, or toxic megacolon) together with laboratory confirmation. All included cases were evaluated using the hospital two-step rapid immunochromatographic algorithm: glutamate dehydrogenase (GDH) antigen screening followed by toxins A/B testing. GDH-positive/toxin-negative specimens did not fulfill the routine laboratory case definition unless alternative confirmatory evidence of pseudomembranous colitis was documented; patients without confirmatory evidence were excluded.

CDI cases were classified according to the ECDC surveillance definitions as healthcare-associated (HA-CDI), community-associated (CA-CDI), or of unknown association (UA-CDI), based on the timing and location of symptom onset in relation to healthcare exposure. HA-CDI was defined as CDI with symptom onset on or after day 3 of hospitalization, or within four weeks after discharge from a healthcare facility, including cases presenting in the community or on days 1–2 of a subsequent hospitalization. CA-CDI was defined as CDI with symptom onset outside a healthcare facility and no discharge from a healthcare facility during the preceding 12 weeks, or onset on days 1–2 of hospitalization in a patient without residence in a healthcare facility during the preceding 12 weeks. Cases with symptom onset outside a healthcare facility 4–12 weeks after discharge, or onset on days 1–2 of hospitalization in patients with healthcare exposure 4–12 weeks earlier, were classified as having an unknown association, according to the ECDC criteria [5].

For the purposes of the present study, HA-CDI cases were further categorized according to the presumed healthcare setting of acquisition as external HA-CDI, when the relevant healthcare exposure occurred in another healthcare facility, and internal HA-CDI, when CDI was presumed to have been acquired during hospitalization at the study hospital. This study-specific subcategorization was based on the patient’s documented hospitalization, transfer, and discharge history and was used to characterize the epidemiological setting of acquisition.

Recurrent CDI (rCDI) was defined as recurrence of symptoms with a positive diagnostic test after resolution of the previous episode. In the absence of molecular typing, recurrent episodes were not further classified as relapse or reinfection [6,7,8].

### 2.4. Data Collection

The date of hospital admission for the index CDI episode was defined as the index date (time zero) for the 90-day follow-up. Demographic and clinical information available during the index hospitalization was extracted from the source records. ATLAS components were assessed using parameters recorded at CDI diagnosis. Urinary-catheter exposure was analyzed separately as an index-hospitalization exposure because reliable catheter-placement timing relative to CDI diagnosis was not available for every patient.

Concomitant infection was defined as a clinically diagnosed infection other than CDI documented during the index hospitalization and supported, when applicable, by microbiological, virological, imaging, or other diagnostic evidence recorded by the treating team. Colonization or screening positivity without compatible clinical findings was recorded separately and was not counted as a concomitant infection. For *Candida albicans*, oral, urinary, and vulvovaginal detections were reviewed according to anatomical site and clinical manifestations. Symptomatic candidiasis requiring treatment comprised oral candidiasis with white plaques and oral pain, symptomatic urinary candidiasis with dysuria and fever, and vulvovaginal candidiasis with pruritus and pain. *Candida* detection without compatible symptoms was not classified as active candidiasis. For the patient-level prevalence analysis, each patient was assigned to a single documented concomitant-infection category; therefore, these categories were mutually exclusive.

Bacterial identification was performed using conventional culture and biochemical methods, while the VITEK 2 automated system (bioMérieux, Marcy-l’Étoile, France) was used for patients with immunosuppression, severe infections, or risk factors for multidrug-resistant pathogens. Antimicrobial susceptibility testing was performed using the VITEK 2 system in these selected cases and by the Kirby–Bauer disk diffusion method in other patients. Minimum inhibitory concentrations (MICs), where available, and inhibition zone diameters were interpreted according to the Clinical and Laboratory Standards Institute (CLSI) M100 Performance Standards for Antimicrobial Susceptibility Testing, 34th edition (2024) [13]. These methods were applied only to isolates from concomitant infections and MDR screening; CDI was diagnosed exclusively with the two-step GDH antigen/toxin A/B algorithm described in Section 2.3.

Indwelling urinary catheter (UC) exposure was recorded as a binary variable (yes/no) for the index hospitalization. The retrospective records did not reliably establish catheter presence at the CDI diagnosis time point for every patient. Accordingly, UC exposure was not interpreted as a baseline causal predictor; its association with 90-day mortality is presented as exploratory and may reflect severity, frailty, immobility, or subsequent clinical management.

The ATLAS score and the Age-adjusted Charlson Comorbidity Index (ACCI) were calculated for each patient using their respective validated methods [14,15]. The ATLAS score incorporates age, systemic antibiotic treatment, leukocyte count, serum albumin concentration, and serum creatinine concentration and was calculated using parameters recorded at CDI diagnosis to assess disease severity and prognosis [14]. The ACCI incorporates age and a weighted set of comorbid conditions and was used to quantify the baseline comorbidity burden and its prognostic impact on mortality [15,16,17,18,19].

All data were anonymized prior to analysis in accordance with institutional confidentiality requirements and applicable personal data protection regulations.

### 2.5. Study Outcome

The primary outcome was 90-day all-cause mortality, defined as death from any cause occurring during the index hospitalization or within 90 days after hospital admission for the index CDI episode (index date/time zero). The study did not adjudicate CDI-attributable mortality; therefore, all mortality analyses refer to all-cause mortality.

Patients discharged alive were followed to day 90 after hospital admission for the index CDI episode to ascertain vital status. When vital status could not be established from hospital records, telephone contact was attempted up to three times with the patient or, when necessary, a relative, solely to determine whether the patient was alive or deceased. Of 191 patients eligible for 90-day follow-up, 21 lacked documented written informed consent authorizing participation/data use and were excluded, leaving 170 patients for analysis. Of these, three died during the index hospitalization and 167 were discharged alive.

### 2.6. Statistical Analysis

Statistical analyses were performed using XLSTAT (version 4.5.2022; Addinsoft, Paris, France).

Categorical variables were summarized as frequencies and percentages, with 95% confidence intervals for key prevalence estimates. The distribution of continuous variables was assessed using the Shapiro–Wilk test together with visual inspection of histograms and Q-Q plots. Continuous variables were summarized as mean ± standard deviation (SD) when approximately normally distributed and as median with interquartile range (IQR) when non-normally distributed. Categorical comparisons used the chi-square test or Fisher’s exact test, as appropriate.

Continuous variables were compared using Student’s *t*-test or one-way analysis of variance (ANOVA) when parametric assumptions were satisfied and the Mann–Whitney U test for non-normally distributed variables, as appropriate. Correlations between continuous variables were assessed using Spearman’s rank correlation coefficient when appropriate.

For binomial proportions reported in the descriptive results, 95% confidence intervals were calculated using the exact Clopper-Pearson method. Associations with 90-day all-cause mortality were assessed using binary logistic regression. Candidate variables were selected on clinical grounds and univariable evidence while avoiding simultaneous inclusion of composite scores and their component variables. The final model contained five parameters and 55 mortality events, corresponding to approximately 11 outcome events per modeled parameter, a ratio chosen to limit overfitting in this modest single-center cohort. Adjusted odds ratios (aORs) with 95% CIs are reported. Model discrimination was assessed by the area under the receiver operating characteristic curve (AUC), with 95% CI estimated by the DeLong method. Because urinary-catheter timing relative to CDI diagnosis was incompletely resolved, the UC association is interpreted as exploratory rather than as a strictly baseline predictive effect. All tests were two-sided and *p* < 0.05 was considered statistically significant.

## 3. Results

A total of 170 patients were included in the analysis.

### 3.1. Demographic Characteristics

Patient age ranged from 18 to 96 years, with a median age of 70 years (IQR, 60–78.5). Overall, 59.4% of patients were older than 65 years and 28.6% were older than 80 years.

The study population comprised 83 men (49.0%) and 87 women (51.0%).

Overall, 72.9% of patients (*n* = 124) were from urban areas, whereas 27.1% (*n* = 46) were from rural areas.

No significant difference in age was observed between male and female patients (*p* = 0.800). Similarly, no significant association was found between sex and place of residence.

Length of hospital stay ranged from 1 to 30 days, with a median of 7 days (IQR, 5–9). Overall, 54.6% of patients were hospitalized for 1–5 days, 38.6% for 6–10 days, and 11 patients (6.5%) for more than 10 days.

### 3.2. Epidemiological Characteristics

Most patients were hospitalized with a first CDI episode (*n* = 144, 84.7%), whereas 26 patients had recurrent CDI (15.3%; 95% CI, 10.2–21.6%). Recurrence status was classified independently of the epidemiological category of the current episode.

Based on the epidemiological investigation, 66 cases (38.8%; 95% CI, 31.5–46.6%) were classified as community-associated CDI, 45 (26.5%; 95% CI, 20.0–33.8%) as external healthcare-associated CDI, and 18 (10.6%; 95% CI, 6.4–16.2%) as internal healthcare-associated CDI. In 41 cases (24.1%; 95% CI, 17.9–31.3%), the epidemiological association could not be reliably established and the cases were classified as CDI of unknown association. These four mutually exclusive categories sum to 170 patients (100%).

The ACCI ranged from 0 to 13, with a median score of 6 and a mean score of 5.8 ± 3.29. Overall, 58.2% of patients (99/170; 95% CI, 50.4–65.7%) had an ACCI score >5.

**Figure 2:**
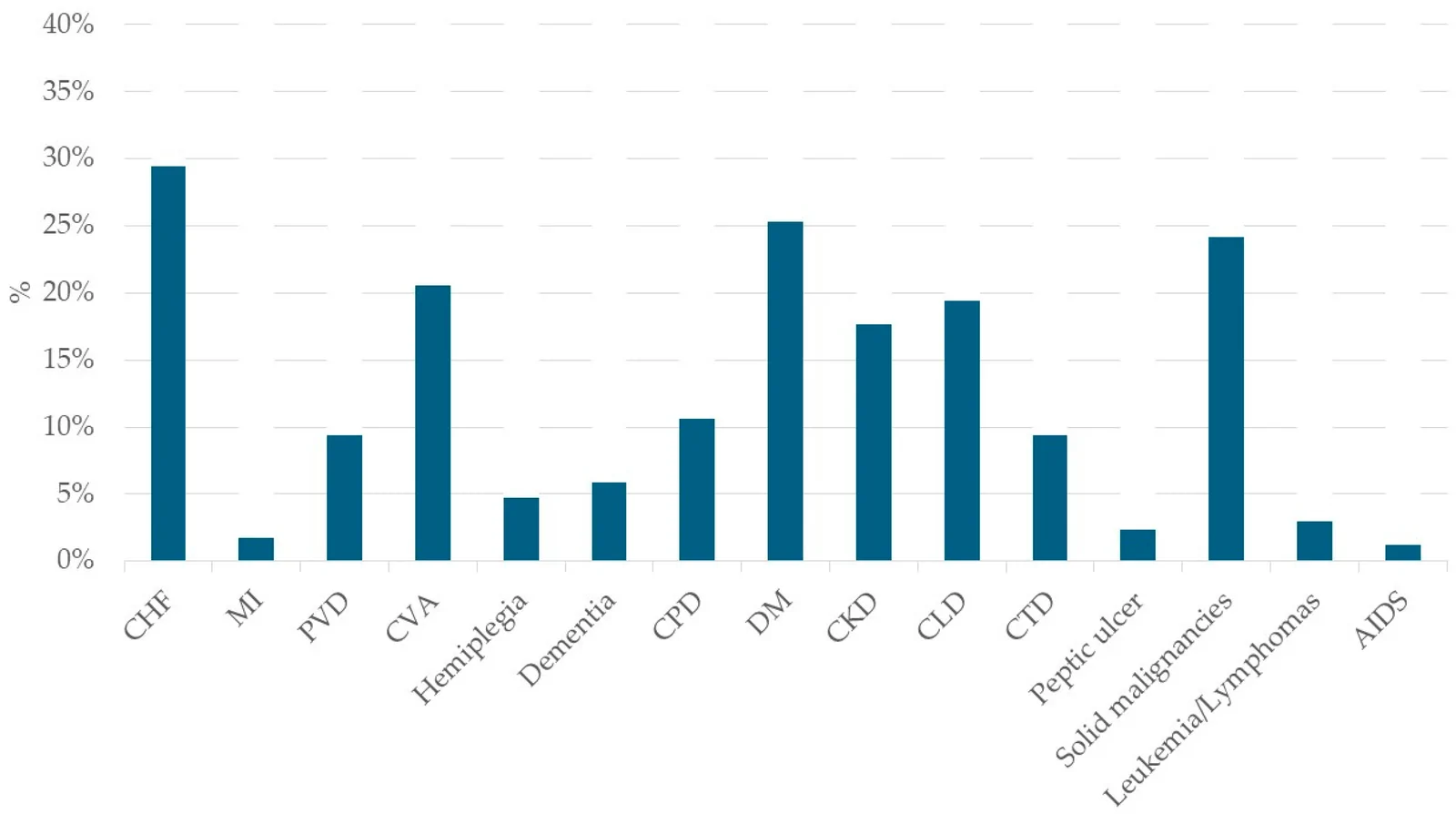
Prevalence of comorbidities included in the age-adjusted Charlson Comorbidity Index (ACCI) among 170 patients. CHF, chronic heart failure; MI, myocardial infarction; PVD, peripheral vascular disease; CVA/TIA, cerebrovascular accident/transient ischemic attack; CPD, chronic pulmonary disease; CTD, connective tissue disease; CLD, chronic liver disease; DM, diabetes mellitus; CKD, chronic kidney disease; AIDS, acquired immunodeficiency syndrome.

Comorbidities were stratified according to their prevalence. The most common comorbidities (prevalence >20%) were chronic cardiac disease (29.4%), diabetes mellitus (25.4%), solid malignancies (22.9%), and cerebrovascular disease (20.5%). Comorbidities with a prevalence of 10–20% included chronic liver disease (19.4%), chronic kidney disease (18.0%), and chronic pulmonary disease (10.5%). Connective tissue diseases (9.4%) and dementia (5.9%) showed intermediate prevalence (5–10%), whereas all remaining comorbidities were identified in fewer than 5% of patients (Figure 2).

### 3.3. Clinical Characteristics

The interval from symptom onset to hospital admission ranged from 1 to 30 days and was non-normally distributed; it is therefore reported as a median of 4 days (IQR, 2.25–7). Overall, 63.0% of patients were admitted within 7 days of symptom onset.

The number of bowel movements ranged from 3 to 15 per day and was non-normally distributed; it is therefore reported as a median of 5 stools/day (IQR, 4–7). Fever (>38 °C) was documented in 16.0% of patients.

The most common documented clinical risk factors potentially associated with gut microbiota disruption included age >65 years (59.4%), recent exposure to broad-spectrum antibiotics (49.4%), malignancy (27.1%), and indwelling UC exposure (16.4%).

Previous antibiotic exposure before CDI was documented in 84/170 patients (49.4%; 95% CI, 41.7–57.2%). Because some patients received more than one antibiotic class, class-specific percentages are non-mutually exclusive and may sum to >100%.

### 3.4. Concomitant Infections and MDR Colonization

Concomitant infections were identified in 95/170 patients (55.9%; 95% CI, 48.1–63.5%). *Candida albicans* was detected in 83 site-specific specimens: oral cavity (*n* = 49), urine (*n* = 15), stool (*n* = 12), and vulvovaginal specimens (*n* = 7). Of the 49 oral detections, 29 patients had symptomatic oral candidiasis characterized by white plaques and oral pain and required treatment. Of the 15 urinary detections, 6 patients had symptomatic urinary candidiasis with dysuria and fever and were treated. All 7 vulvovaginal detections represented symptomatic vulvovaginal candidiasis with pruritus and pain and were treated. Stool detection and other *Candida* detections without compatible clinical manifestations were not classified as active candidiasis. Thus, only symptomatic, treated candidiasis (*n* = 42) was counted as a concomitant infection. Other documented concomitant infections included COVID-19 (*n* = 3), influenza A (*n* = 1), influenza B (*n* = 1), HIV infection (*n* = 3), hepatitis C virus infection (*n* = 2), hepatitis B virus infection (*n* = 6), and tuberculosis (*n* = 2). Eighteen bacterial infections were documented, including *Escherichia coli* (*n* = 5), *Klebsiella pneumoniae* (*n* = 5), *Proteus* spp. (*n* = 1), *Salmonella* spp. (*n* = 1), *Acinetobacter baumannii* (*n* = 1), *Pseudomonas aeruginosa* (*n* = 1), *Streptococcus gallolyticus* (*n* = 1), *Streptococcus pyogenes* (*n* = 1), *Staphylococcus aureus* (*n* = 1), and *Enterococcus* spp. (*n* = 1). Additional clinically diagnosed concomitant infections without a microbiological isolate included pneumonia (*n* = 7), urinary tract infection (*n* = 7), and skin and soft-tissue infection (*n* = 3). With this classification, each of the 95 patients had one documented concomitant infection, and the infection categories used to derive the patient-level prevalence were mutually exclusive. Concomitant antibiotic therapy for infections other than CDI was administered to 41 patients (24.1%).

Rectal screening for multidrug-resistant (MDR) organisms was performed in 22 patients transferred from other hospital departments or healthcare facilities. Positive screening results were obtained in 10 patients (45.5%), identifying MDR *Escherichia coli* (*n* = 4), MDR *Klebsiella pneumoniae* (*n* = 3), vancomycin-resistant *Enterococcus* (VRE; *n* = 1), and methicillin-resistant *Staphylococcus aureus* (MRSA; *n* = 1).

### 3.5. Disease Severity

Disease severity was assessed using the ATLAS score. For the mortality analysis, patients were categorized according to the prespecified cutoff as ATLAS >4 (43/170, 25.3%) and ATLAS ≤4 (127/170, 74.7%).

### 3.6. Clinical Management and In-Hospital Outcomes

Oral vancomycin was the standard antimicrobial therapy for CDI and was administered at 125 mg every 6 h for first episodes, with higher doses used when clinically indicated. Intravenous metronidazole (500 mg every 8 h) was added in selected patients with severe, fulminant, or complicated disease. The documented duration of vancomycin therapy during hospitalization ranged from 1 to 18 days, with a mean duration of 7 ± 2.4 days. This represents the in-hospital treatment period only, because some patients had initiated therapy before admission or continued treatment after discharge; total treatment duration could therefore not be reliably assessed from the available records. Major in-hospital complications included sepsis in 7/170 patients (4.1%; 95% CI, 1.7–8.3%) and toxic megacolon in 4/170 (2.4%; 95% CI, 0.6–5.9%); 3/170 patients died during hospitalization (1.8%; 95% CI, 0.4–5.1%).

### 3.7. Ninety-Day Mortality and Its Predictors

Overall, 55/170 patients died within 90 days after hospital admission for the index CDI episode (32.4%; 95% CI, 25.4–39.9%). Three deaths occurred during the index hospitalization; among 167 patients discharged alive, 52 died during follow-up.

In univariable analysis, chronic kidney disease, dementia, low serum albumin, healthcare-associated CDI, indwelling urinary-catheter exposure, ACCI >5, and ATLAS score >4 showed statistically significant associations with 90-day all-cause mortality (Table 1). For HA-CDI, 30 of 63 patients died and 33 survived; among patients without HA-CDI, 25 died and 82 survived, corresponding to an unadjusted OR of 2.98 (95% CI, 1.53–5.81; *p* = 0.001). Malignancy and diabetes did not meet the prespecified *p* < 0.05 threshold and were not described as statistically significant.

**Figure 3:**
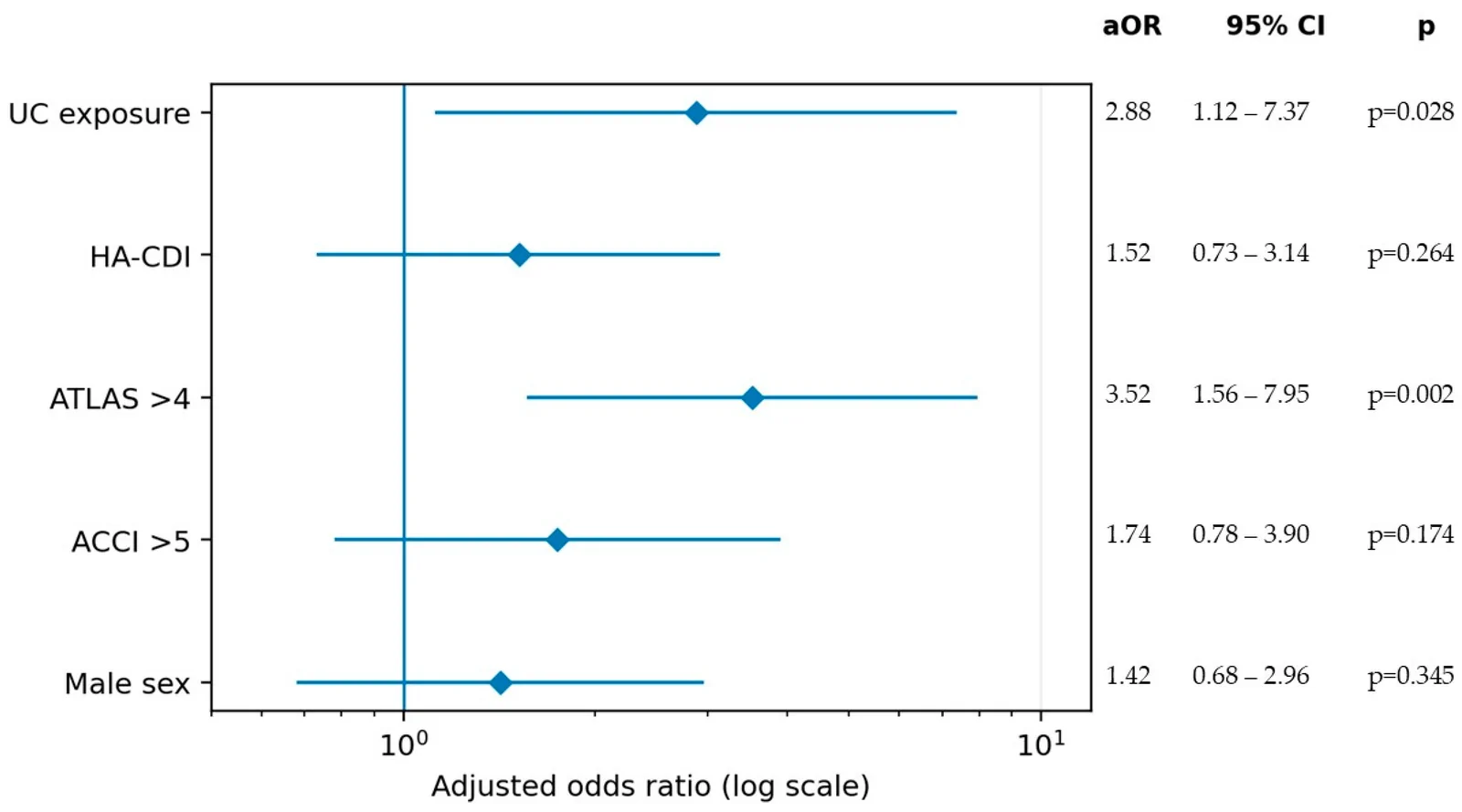
Adjusted associations of the five variables included in the final multivariable logistic regression model with 90-day all-cause mortality. Points indicate adjusted odds ratios (aORs) and horizontal bars indicate 95% confidence intervals. The vertical reference line denotes an odds ratio of 1. The model included ATLAS score >4, urinary-catheter exposure, HA-CDI, ACCI >5, and sex. The urinary-catheter estimate should be interpreted as exploratory because exposure timing relative to CDI diagnosis was not reliably available for every patient. HA-CDI, healthcare-associated CDI; ACCI, age-adjusted Charlson Comorbidity Index.

In the final multivariable logistic regression model, ATLAS score >4 was associated with higher odds of 90-day all-cause mortality (aOR, 3.52; 95% CI, 1.56–7.95; *p* = 0.002). Indwelling UC exposure during the index hospitalization was also associated with mortality (aOR, 2.88; 95% CI, 1.12–7.37; *p* = 0.028). The model was re-run with the corrected HA-CDI variable; HA-CDI was not independently associated with 90-day mortality (aOR, 1.52; 95% CI, 0.73–3.14; *p* = 0.264). Because UC timing relative to CDI diagnosis was not reliably available for every patient, this association is exploratory and does not establish a baseline predictive or causal effect. ACCI >5 and sex were not independently associated with mortality after adjustment [Figure 3].

The final multivariable model showed good discriminatory ability for 90-day mortality, with an area under the receiver operating characteristic curve (AUC) of 0.746 (95% CI, 0.664–0.828; DeLong method, *p* < 0.001).

## 4. Discussion

### 4.1. Principal Findings

In this retrospective observational study, we evaluated the clinical and epidemiological characteristics of hospitalized patients with CDI and investigated factors associated with 90-day all-cause mortality. The main findings were the substantial 90-day mortality burden and the adjusted associations of ATLAS score >4 and urinary-catheter exposure with mortality.

An ATLAS score >4 was associated with mortality after adjustment, consistent with the score’s integration of age, systemic antibiotic exposure, leukocyte count, albumin, and creatinine. Urinary-catheter exposure was also associated with mortality; however, because catheter timing relative to CDI diagnosis was not consistently available, it is best interpreted as a marker of clinical vulnerability or care complexity rather than a causal or baseline prognostic factor.

The demographic and epidemiological characteristics of our cohort were consistent with the profile commonly described among hospitalized patients with CDI. The cohort was predominantly composed of older adults (median age, 70 years; 59.4% aged >65 years) and was characterized by a high burden of comorbidity, with 58.2% having an age-adjusted Charlson Comorbidity Index (ACCI) >5. Nearly half of the patients (49.4%) had a history of prior antibiotic exposure, most frequently involving cephalosporins and penicillins. These findings are consistent with the established epidemiology of CDI, in which advanced age, multimorbidity, and antimicrobial exposure represent important determinants of susceptibility and adverse outcomes.

The final multivariable model identified ATLAS >4 and indwelling UC exposure as variables associated with 90-day all-cause mortality after adjustment, whereas HA-CDI (aOR, 1.52; 95% CI, 0.73–3.14; *p* = 0.264), ACCI >5, and sex were not independently associated with the outcome. The prognostic relevance of ATLAS is biologically plausible because the score integrates several parameters reflecting both acute disease severity and host vulnerability. In contrast, the lack of an independent association between ACCI >5 and mortality suggests that the prognostic information provided by global comorbidity burden may overlap with measures of acute illness and functional vulnerability. The final model demonstrated good discriminatory ability for 90-day mortality, with an AUC of 0.746 (95% CI, 0.664–0.828; DeLong test, *p* < 0.001).

### 4.2. Comparison with Previous Studies

The demographic characteristics of our cohort are broadly consistent with contemporary European epidemiological data. The predominance of older patients and the high burden of multimorbidity are in line with findings from Germany, Spain, and Sweden [20,21,22,23], while the high prevalence of previous antibiotic exposure is consistent with the established role of antimicrobial-driven intestinal dysbiosis [24,25,26]. Recent Romanian studies provide additional context. Vasile et al. reported contemporary CDI epidemiology and substantial previous antibiotic exposure in a tertiary infectious diseases setting in Bucharest [9]. Stămăteanu et al. documented the burden and outcomes of healthcare-associated CDI in north-eastern Romania [27], whereas Stoian et al. described the complexity of CDI among critically ill patients in Târgu Mureș, particularly in the setting of SARS-CoV-2 coinfection [28]. Differences in populations, settings, and outcome definitions preclude direct numerical comparison, but these studies demonstrate the heterogeneous clinical and epidemiological profile of CDI across Romanian healthcare settings. Our study extends this evidence by focusing on 90-day all-cause mortality in a regional infectious diseases hospital in south-eastern Romania.

The substantial mortality observed during the 90-day follow-up underscores the importance of evaluating outcomes beyond the index hospitalization. Most deaths occurred after discharge rather than during the acute admission, indicating that in-hospital mortality alone would underestimate the short-term mortality burden in this cohort.

The association between ATLAS score >4 and 90-day mortality is consistent with previous evaluations of prognostic models in CDI [29,30,31,32]. ATLAS combines acute physiological and host-vulnerability measures, which provides biological plausibility for its association with adverse outcomes. In our cohort, ATLAS >4 was associated with higher adjusted odds of 90-day all-cause mortality (aOR, 3.52; 95% CI, 1.56–7.95). Given the single-center design and modest sample size, this estimate should be viewed as an internally observed association requiring external validation rather than as a validated threshold for clinical decision-making.

Urinary-catheter exposure was also associated with 90-day mortality, but this finding requires particular caution. Catheterization may reflect frailty, functional dependence, urinary retention, immobility, severity of illness, or exposure to invasive care. Moreover, the retrospective records did not reliably establish whether catheter placement preceded CDI diagnosis in every patient. The association therefore cannot be interpreted as evidence that catheterization causes mortality or as a strictly baseline predictive effect; residual confounding and time-dependent exposure are plausible explanations [33,34,35,36,37].

Interestingly, ACCI >5 was not independently associated with 90-day mortality in the final multivariable model despite reflecting the substantial comorbidity burden of our cohort. This finding may indicate that the prognostic contribution of chronic comorbidities is partly captured by measures of acute disease severity and functional vulnerability. Importantly, however, this should not be interpreted as evidence that comorbidities are clinically irrelevant. Rather, their individual prognostic effects may overlap with other variables included in the model.

Albumin and dementia were associated with mortality in preliminary analyses but were not retained as individual predictors in the final model because albumin is a component of the ATLAS score and dementia contributes to the Charlson Comorbidity Index. Including these variables alongside their respective composite scores could result in redundancy and double counting of prognostic information. Therefore, the final model retained ATLAS and ACCI scores as composite measures.

### 4.3. Clinical Implications

Our findings emphasize the importance of assessing outcomes beyond hospital discharge in patients with CDI. Although only three patients died during hospitalization, an additional 52 deaths occurred during the subsequent 90-day follow-up, indicating that hospital mortality alone would substantially underestimate the mortality burden observed in this cohort. The high proportion of deaths occurring after discharge highlights the vulnerability of patients recovering from CDI and the potential contribution of recurrent infection, persistent disruption of the intestinal microbiota, functional decline, and decompensation of underlying chronic conditions to longer-term outcomes [22,38,39,40].

The observed associations may help generate hypotheses for future prospective risk-assessment studies, but they do not by themselves justify implementation of a new clinical risk-stratification pathway. ATLAS is readily available at diagnosis and may be useful for describing illness severity. Urinary-catheter exposure should prompt routine reassessment of catheter necessity according to established infection-prevention practice, but our data do not show that catheter removal would reduce mortality.

The substantial proportion of deaths occurring after discharge also supports a broader approach to CDI management that extends beyond treatment of the acute infection. Structured follow-up of high-risk patients, early recognition of recurrence, optimization of comorbid conditions, appropriate antimicrobial use, and measures aimed at reducing avoidable healthcare-associated complications may be relevant components of post-discharge care [41,42,43,44,45,46,47,48]. These strategies may be particularly important in settings where elderly and multimorbid patients are discharged with persistent functional limitations or a high need for healthcare support.

### 4.4. Strengths of the Study

The present study has several strengths. First, CDI cases were diagnosed using a standardized two-step laboratory algorithm within a single institution, reducing diagnostic heterogeneity. Second, the study included systematic 90-day follow-up from hospital admission for the index CDI episode, allowing assessment of mortality beyond the index hospitalization. Third, detailed clinical and epidemiological data allowed simultaneous assessment of acute disease severity, comorbidity burden, healthcare-associated factors, and markers of clinical vulnerability. Finally, the multivariable analysis avoided simultaneous inclusion of composite scores and their component variables, reducing redundancy.

### 4.5. Limitations

This study has several limitations. First, its retrospective single-center design limits causal inference and external generalizability. Twenty-one of 191 patients eligible for 90-day follow-up (11.0%) lacked documented written informed consent authorizing participation/data use and were excluded, leaving 170 patients for analysis. The final multivariable model contained five parameters and 55 mortality events (approximately 11 events per parameter), which is acceptable for an exploratory model but still provides limited precision and does not eliminate overfitting risk. Second, urinary-catheter timing relative to CDI diagnosis was not reliably available for every patient, so catheter exposure cannot be interpreted as a baseline predictor and may be affected by time-dependent exposure and confounding by illness severity or frailty. Third, the primary outcome was 90-day all-cause rather than CDI-attributable mortality, and causes of post-discharge death were not adjudicated. Fourth, molecular strain typing was not performed, precluding distinction between relapse and reinfection. Fifth, concomitant-infection documentation was retrospective; *Candida* findings were therefore separated into symptomatic treated candidiasis and detection without compatible clinical manifestations. For the patient-level prevalence analysis, each patient was assigned to one documented concomitant-infection category. Sixth, the hospital is a specialized regional infectious diseases center in Galați; differences in case mix, referral patterns, stewardship, discharge planning, and follow-up limit generalizability beyond this institution and south-eastern Romania. Finally, model calibration and clinical utility were not formally evaluated; prospective multicenter external validation is required before clinical implementation.

### 4.6. Future Research

Prospective multicenter studies involving larger and more diverse patient populations are needed to externally validate the prognostic value of an ATLAS score >4 and indwelling urinary catheter use for 90-day mortality in patients with CDI. Future studies should also evaluate model calibration and clinical utility to determine whether these variables can be integrated into a reliable clinical risk-stratification approach.

Further research should distinguish between all-cause and CDI-attributable mortality and systematically assess causes of death during follow-up. Molecular strain typing would also be valuable for differentiating relapse from reinfection among patients with recurrent CDI and for clarifying their respective contributions to longer-term outcomes.

Finally, interventional studies should evaluate potentially modifiable factors identified through observational research. Studies assessing structured post-discharge follow-up, early recognition and management of recurrent CDI, and systematic reassessment of the need for indwelling urinary catheters could determine whether these strategies improve longer-term outcomes. Such studies would help establish whether the associations observed in the present study represent clinically modifiable risk factors rather than markers of underlying patient vulnerability.

## 5. Conclusions

In this single-center cohort from Galați, Romania, 90-day all-cause mortality among hospitalized patients with CDI was substantial. An ATLAS score >4 was independently associated with 90-day mortality, whereas a high comorbidity burden, as assessed by the ACCI, and healthcare-associated CDI were not independently associated with the outcome after adjustment. Indwelling urinary catheter use was also associated with mortality after adjustment; however, because the timing of catheter placement relative to CDI diagnosis was not reliably available, this association should be considered exploratory and may reflect underlying frailty, functional dependence, greater illness severity, or healthcare exposure rather than a causal effect. These findings suggest that acute disease severity and markers of clinical and functional vulnerability may provide prognostic information beyond baseline comorbidity burden. The present study does not validate a new clinical prediction tool. Further prospective, multicenter studies are needed to externally validate these findings and to determine whether structured post-discharge follow-up and interventions addressing potentially modifiable factors can improve outcomes.

## Figures and Tables

**Table 1:** Univariable associations with 90-day all-cause mortality in patients with CDI. ORs compare the first category shown with the second category as the reference. CI, confidence interval; OR, odds ratio; CHF, chronic heart failure; CKD, chronic kidney disease; CVD, cerebrovascular disease; HA-CDI, healthcare-associated CDI; UC, urinary-catheter exposure; ACCI, age-adjusted Charlson Comorbidity Index.

Variable	Category	Survivors	90-day Deaths	OR	95% CI	*p*
Sex	Male	50	32	1.81	0.94–3.47	0.100
	Female	65	23			
Living	Urban	83	41	1.13	0.54–2.35	0.854
	Rural	32	14			
CHF	Yes	29	21	1.83	0.92–3.64	0.105
	No	86	34			
CVD	Yes	21	14	1.53	0.71–3.30	0.313
	No	94	41			
Diabetes	Yes	25	20	2.06	1.02–4.17	0.062
	No	90	35			
CKD	Yes	16	16	2.54	1.16–5.57	0.022
	No	99	39			
Dementia	Yes	3	7	5.44	1.35–21.95	0.014
	No	112	48			
Malignancy	Yes	29	22	1.98	1.00–3.92	0.073
	No	86	33			
Albumin (g/dL)	≤3	34	29	2.66	1.37–5.16	0.004
	>3	81	26			
HA-CDI	Yes	33	30	2.98	1.53–5.81	0.001
	No	82	25			
UC	Yes	11	16	3.88	1.66–9.09	0.003
	No	104	39			
ACCI	>5	57	42	3.29	1.60–6.76	<0.001
	≤5	58	13			
ATLAS score	>4	17	26	5.17	2.47–10.82	<0.001
	≤4	98	29			

Note: *p* values were calculated using two-sided Fisher’s exact test. CI, confidence interval; OR, odds ratio; CHF, chronic heart failure; CKD, chronic kidney disease; CVD, cerebrovascular disease; HA-CDI, healthcare-associated CDI; UC, urinary-catheter exposure; ACCI, age-adjusted Charlson Comorbidity Index.

## Data Availability

The data supporting the findings of this study are presented within the article. Individual participant data are not publicly available due to privacy and ethical restrictions. Additional information may be obtained from the corresponding authors upon reasonable request.

## Abbreviations

The following abbreviations are used in this manuscript:

ACCI	Age-adjusted Charlson Comorbidity Index
CDI	*Clostridioides difficile* infection
CA-CDI	community-associated *Clostridioides difficile* infection
CHF	Chronic heart failure
CKD	Chronic kidney disease
CLD	Chronic liver disease
CPD	Chronic pulmonary disease
CTD	Connective tissue disease
CVA/TIA	Cerebrovascular accident/transient ischemic attack
DM	Diabetes mellitus
ECDC	European Centre for Disease Prevention and Control
HA-CDI	Healthcare-associated *Clostridioides difficile* infection
HAIs	Healthcare-associated infections
MDR	Multidrug-resistant
MI	Myocardial Infarction
OR	odds ratio
PVD	Peripheral vascular disease
rCDI	recurrent *Clostridioides difficile* infection
UC	urinary-catheter exposure
WHO	World Health Organization
